# Medulla oblongata transcriptome changes during presymptomatic natural scrapie and their association with prion-related lesions

**DOI:** 10.1186/1471-2164-13-399

**Published:** 2012-08-16

**Authors:** Hicham Filali, Inmaculada Martin-Burriel, Frank Harders, Luis Varona, Carmen Serrano, Cristina Acín, Juan J Badiola, Alex Bossers, Rosa Bolea

**Affiliations:** 1 Centro de Investigación en Encefalopatías y Enfermedades Transmisibles Emergentes. Facultad de Veterinaria, Universidad de Zaragoza, Zaragoza, Spain; 2 Laboratorio de Genética Bioquímica (LAGENBIO), Facultad de Veterinaria, Universidad de Zaragoza, Zaragoza, Spain; 3 Instituto Aragonés de Ciencias de la Salud, Zaragoza, Spain; 4 Central Veterinary Institute of Wageningen UR (CVI), Lelystad, The Netherlands; 5 Unidad de Genética Cuantitativa y Mejora Animal. Facultad de Veterinaria, Universidad de Zaragoza, Zaragoza, Spain

**Keywords:** Natural scrapie, Preclinical sheep, Microarray, Genetic expression, Real time PCR, Prion

## Abstract

**Background:**

The pathogenesis of natural scrapie and other prion diseases is still poorly understood. Determining the variations in the transcriptome in the early phases of the disease might clarify some of the molecular mechanisms of the prion-induced pathology and allow for the development of new biomarkers for diagnosis and therapy. This study is the first to focus on the identification of genes regulated during the preclinical phases of natural scrapie in the ovine medulla oblongata (MO) and the association of these genes with prion deposition, astrocytosis and spongiosis.

**Results:**

A custom microarray platform revealed that 86 significant probes had expression changes greater than 2-fold. From these probes, we identified 32 genes with known function; the highest number of regulated genes was included in the phosphoprotein-encoding group. Genes encoding extracellular marker proteins and those involved in the immune response and apoptosis were also differentially expressed. In addition, we investigated the relationship between the gene expression profiles and the appearance of the main scrapie-associated brain lesions. Quantitative Real-time PCR was used to validate the expression of some of the regulated genes, thus showing the reliability of the microarray hybridization technology.

**Conclusions:**

Genes involved in protein and metal binding and oxidoreductase activity were associated with prion deposition. The expression of glial fibrillary acidic protein (GFAP) was associated with changes in the expression of genes encoding proteins with oxidoreductase and phosphatase activity, and the expression of spongiosis was related to genes encoding extracellular matrix components or transmembrane transporters. This is the first genome-wide expression study performed in naturally infected sheep with preclinical scrapie. As in previous studies, our findings confirm the close relationship between scrapie and other neurodegenerative diseases.

## Background

Scrapie is a prion-associated encephalopathy that occurs naturally in sheep and goats. It is characterized by the accumulation of a pathological agent, the prion protein (PrP^Sc^), mainly in the central nervous system [1]. PrP^Sc^ differs from the endogenous normal form (PrP^c^) in its conformation, partial resistance to proteolytic degradation and insolubility in the presence of detergents [2,3]. Scrapie is included in transmissible spongiform encephalopathies (TSEs), a disease class that also affects humans (e.g., Creutzfeldt-Jakob disease and Kuru) and cattle (e.g., bovine spongiform encephalopathy [BSE]) [4-6].

The incubation period of the disease is long and asymptomatic. PrP^Sc^ can be detected in VRQ/VRQ sheep, genotype for the PRNP gene, two months after infection [7]. Three to six months after infection, the pathological agent is detected in the lymphoid formations associated with the gastrointestinal tract [8,9]. From six to nine months, the secondary lymphoid organs are also infected, and finally, at the tenth month after infection, the central nervous system is affected [10-12].

The neuropathological events in prion diseases occur at different times depending on the disease. High levels of PrP^Sc^ exist without clinical disease in Gerstmann-Sträussler syndrome [13]; conversely, PrP^Sc^ is present in very low levels in fatal familiar insomnia [14]. The degree of prion accumulation in specific brain regions does not correlate with the clinical features (reviewed in [15]). In addition to prion deposition, other molecular mechanisms act early during the disease. For example, the brain undergoes oxidative stress in the early stages of prion invasion into the brain and may predispose the brain to neurodegenerative mechanisms [16]. Genomic analysis confirmed the induction of cellular stress (oxidative stress and ER stress) and the activation of other molecular pathways in a murine model of prion disease [17]. Other functional genomic studies performed in animal models of scrapie infection have indicated that several genes are misregulated in the early phases of the infection [18-24].

To date, very few genomic approaches have focused on the analysis of the early molecular events in prion diseases and, to a lesser extent, studies dealing with the natural disease. The identification of the genes involved in the preclinical changes of the disease can help in the discovery of new biomarkers and targets for future diagnosis tests or treatments. In an earlier published work, we presented the differentially expressed genes in the brains of scrapie-symptomatic sheep and the relationship between scrapie-related neuropathological changes and the transcriptional activities of the identified genes [25]. The objectives of the present study were to identify the genes that are differentially expressed during natural preclinical scrapie infection in sheep using a CVI custom designed 4x44K ovine microarray and to determine the relationship between their expression patterns and prion-associated lesions. In this way, we discuss the variation in gene expression and its association with scrapie neuropathology during the progression of the disease.

## Methods

### Ethics statement

This study was performed in strict accordance with the recommendations for the care and use of experimental animals of the University of Zaragoza, in accordance with the law (R.D. 1201/2005). The protocol was approved by the Committee on the Ethics of Animal Experiments (Permit Number: PI02/08).

### Animals

A total of 10 Rasa Aragonesa female sheep aged 1–5 years were included in this study. Six of them were selected from flocks located in areas free of scrapie and were used as controls.

Four of the animals exhibited preclinical signs of scrapie, and the diagnoses were made by third eyelid biopsies [26] and confirmed using the rapid test (TeEsE, Bio-Rad) and immunohistochemistry to detect PrP^Sc^ using the 6 H4 monoclonal antibody [27]. This characterization was performed considering the presence of the clinical signs associated with the disease as per previously reported criteria [26]. All of the animals belonged to flocks that had been previously characterized as scrapie-affected flocks and were located in different geographical areas. The animals were genotyped for PRNP polymorphisms via full Open Reading Frame sequencing as previously reported [28], and the sheep chosen for this study displayed the ARQ/ARQ genotype without other coding mutations outside the 136, 154 and 171 codons, which is the most susceptible genotype in this ovine breed [28]. The presence of the prion protein was confirmed by immunohistochemical methods and western blotting [27].

### Tissue collection and RNA isolation

Animals were sacrificed by intravenous injection of sodium pentobarbital and exsanguination. Necropsy was performed immediately, and the physical examination of the scrapie-infected and control animals did not reveal any additional pathological signs. The samples were rapidly preserved and processed according to established guidelines regarding safety. The lesion pattern in scrapie is bilateral; therefore, one-half of the caudal medulla oblongata, including the obex, was snap-frozen in liquid nitrogen prior to long-term storage at −80°C until RNA extraction. The other half was formalin-fixed and paraffin-embedded for further histopathological analysis. Total RNA was isolated from a tissuemizer-disrupted medulla oblongata in duplicate using TRIzol® (Invitrogen AG) followed by a phenol and chloroform extraction and subjected to a purification step with the NucleoSpin® RNA clean-up kit RNAII (Macherey-Nagel GmbH & Co. KG). The quality of the total RNA was assessed using the RNA 6000 Nano Assay kit and the 2100 Bioanalyzer (Agilent Technologies). The RNA integrity number (RIN) index for each sample was estimated using the Agilent 2100 Expert software. The RIN provides a numerical assessment of the integrity of RNA that facilitates the standardization of the quality interpretation. Only high quality RNA samples with an RIN number equal to or higher than 7 were further processed for microarray analysis.

### Histology and prion immunohistochemical detection

A histopathological study of the medulla oblongata at the level of the obex was performed in HE-stained slices (one from each individual control and each positive animal).

Immunohistochemical (IHC) studies were performed on adjacent sections. For every antibody, positive and negative controls (the omission of primary antibodies from the control and scrapie slides) were performed.

Detection of the prion proteins was performed following pretreatment as previously described [29]. Briefly, sections were pretreated with 98% formic acid, hydrated and then autoclaved to enhance antigen retrieval. To block endogenous peroxidase activity, the sections were incubated with blocking reagent (DAKO) for 10 min after proteinase K digestion (Roche, 4 g/ml). Next, the sections were incubated with the monoclonal primary antibody L42 (R-Biopharm, dilution 1:500) at RT for 30 min. Endogenous peroxidase blocking was used to process sections. The enzyme-conjugated polymer Envision (DAKO, 30 min) was used as the visualization system and DAB (DAKO, 10 min) as the chromogen. The sections were counterstained with hematoxylin.

Astrocytosis was evaluated based on glial fibrillary acidic protein (GFAP) immunostaining, as previously described [30,31]. Briefly, after heat-induced epitope retrieval pretreatment with citrate buffer (pH 6.0), the sections were incubated for 1 h at RT with a rabbit polyclonal anti-GFAP antibody (DAKO, dilution 1:400). The omission of the primary antibodies from the control and scrapie slides served as negative controls in the routine immunoreactions.

The preparations were examined with a Zeiss Axioskop 40 optical microscope (Carl Zeiss AG) and a 40 × −magnification objective lens (Carl Zeiss AG). The images were captured with a digital camera (AxioCam MRc5, Zeiss AG) that was coupled to the microscope and a computer and were analyzed using the ImageJ 1.4.3.67 image-analysis software package (Psion Image, NIH) to determine the areas occupied by PrP^Sc^ deposition, astrocytosis and spongiosis. For the evaluation of the IHC and HE slides, captured images were opened in NIH Image/ImageJ using the area method to evaluate the indices of positivity. The total area occupied by brown markers (PrP and GFAP) or by white spaces (spongiosis) was estimated by setting a “threshold” using the thresholding tool for the selection of these areas, and the positive IHC/HE index for that image was calculated. Using the Student´s *t* test, significant differences between the control and scrapie groups were detected.

### Custom sheep oligo-DNA microarray

The custom CVI 4x44K microarrays contained custom eArray-designed 60-mer probes on previously sequenced normalized and subtracted cDNA libraries of ovine Peyer's Patch, obex and tonsil, supplemented by the publicly available *Ovis aries* transcripts from the NCBI/EBI databases and by the Agilent *O. aries* transcript catalog. All of the arrays were printed using Sureprint technology (Agilent Technologies).

### Preparation of the labeled cDNA and microarray hybridization

All of the procedures for the preparation of the labeled cRNA probes and subsequent Genechip hybridizations were performed according to the Agilent Technologies One-Color Microarray-Based Gene Expression Analysis guidelines. First, cDNA was synthesized using 1 μg total RNA as a template and the T7 Promoter Primer of Agilent One-Color RNA Spike-In (Quick Amp Kit, One-Color, Agilent Technologies). The cDNA was then transcribed and labeled using T7 RNA Polymerase and cyanine 3-CTP. Finally, the labeled cRNAs were cleaned up using Qiagen RNeasy mini spin columns.

The samples were then hybridized to custom CVI-Agilent 4x44K chips for 17 h at 65°C and 6 rpm. Following the manufacturer’s protocol, the chips were then washed and incubated with wash buffers and scanned using the GenePix 4200AL Scanner (Axon Instruments) in conjunction with GenePix Pro 6.0 software.

The hybridizations of each sample were performed in duplicate, resulting in 8 microarrays for the preclinical scrapie animals and 12 for the negative control animals.

### Microarray data analysis

The hybridization data were extracted with the Agilent Feature Extraction, version 9.5.3.1, image analysis application (Agilent Technologies) before processing with GeneSpring GX 10.0.2 (Agilent Technologies). Using the 75th percentile method intensity, the chip values were normalized, and the expression values were calculated. The global medulla oblongata gene expression profiles from the clinical scrapie-infected animals were compared to the negative controls, through a linear model that accounts for both technical (random animal effects) and biological replicates (disease effects). In addition, a multiple testing correction proposed by Benjamini-Hochberg was applied. Further, only genes with a *P*-value ≤ 0.05 for the difference between healthy and preclinical individuals and a 2-fold change (FC) as the lower limit were selected. Although 2-FC is used as cutoff value, according to our experience, we consider that the major conclusion of our work is not changing even if we would have used a different threshold, but always higher than 1.5. These genes were clustered by their Euclidean distance coefficient using the PermutMatrix software [32]. A BLAST search of the GenBank database was performed to identify the genes that were similar to the differentially expressed probes. The molecular functions of the genes were classified according to Gene Ontology (GO) using an on-line functional annotation of DAVID Bioinformatics Resources 2008 [33,34] (NIAID/NIH, USA).

### The relationship between neuropathology and gene expression

Using a Mixed Model Analysis under a Bayesian approach by the Gene Expression Analysis with Mixed Models (GEAMM) software [35], the relationship between neuropathological lesions and gene expression was analyzed. The statistical model assumed the following Bayesian likelihood of logarithm of gene expression data provided by the oligo-DNA microarray: $p\left(y|a,b,R\right)\sim N\left(Xa+cb,I⊗R\right)$, where *a* is the array effect, *b* is the vector regression slope associated with the numerical valuation of the neuropathological changes (*c*: prion deposition, spongiosis or astrocytosis), X is the incidence matrix that relates the array effects to the logarithm of gene expression data (y), and R is the matrix of residual (co)variances with probe-specific residual variance and null residual covariances. Prior distributions were assumed to be flat for *a*, *b* and R. A more detailed description of the statistical procedure was described by Casellas et al. (2008) [35].

The Bayesian analysis was performed using a Gibbs sampler approach [36] with a single chain of 500,000 iterations after discarding the first 50,000. The results with a posterior probability below 0.01 for a regression slope associated with a neuropathological lesion greater (or lower) than zero were selected.

### Quantitative real-time PCR

Quantitative real-time PCR (qRT-PCR) was performed to confirm the expression of the 12 genes/sequences involved in the mechanisms related to neurodegenerative or reparation processes and/or had a high level of differential expression in the scrapie group compared to the controls in the oligo-DNA microarray expression analysis. Eight of these genes also displayed the highest significance in the Mixed Model Analysis. The PCR primer sequences used for the quantification of the genes encoding amyloid beta (A4) precursor (*APP*), aquaporin 4 (*AQP4*)*,* calcineurin-like phosphoesterase domain-containing 1 (*CPPED1*)*,* golgi golgin subfamily 4 (*GOLGA4*), maguk p55 subfamily member 7 (*MPP7*), nell2 (*NELL2*), CD3 gamma chain (*CD3G*), granulysin (*GNLY*), lysosomal protein transmembrane 4 beta (*LAPTM4B*) and serine/arginine-rich splicing factor 3 (*SRSF3*) and the two ovine scrapie related sequences (OSRS1) and (OSRS2) are shown in Table 1. RNA samples used for qRT-PCR were the same used for microarray experiments, the qRT-PCR assays were designed with Primer Express 2.0 software (Applied Biosystems) to select appropriate primer sequences from known sheep or bovine sequences. Whenever possible, the exon-exon border was included to prevent the amplification of genomic DNA in the PCR reaction. Complementary DNA (cDNA) was synthesized from 1 μg RNA using random hexamer primers with the Superscript First Standard Synthesis System for RT-PCR (Invitrogen). To confirm the elimination of any remaining DNA, reverse transcription with and without the enzyme was performed.

**Table 1:** Genes analyzed by quantitative real-time PCR

	**Gene**	**Primer sequence**	**Size (bp)**	**Accession number (GenBank)**
Upregulated genes/sequences	APP	F: ACCCCTGACGCCGTTGAC	121	NM_001076796.1
		R: TCATGACCTGGGACATCCTCTC		
	AQP4	F: GTTCACGGAAATCTTAGCGCT	104	NM_181003.2
		R: TCAGTCCGTTTGGAATCACAG		
	CPPED1	F: TTGGATGGCATCACCGACTT	101	NM_001031771.2
		R: TTTGCGACCTCATGAACCAC		
	GOLGA4	F: TCTACCAAAACCACTGCCTCAA	88	NM_001192125.1
		R: TCCCACTACTGGCTCTACATCACT		
	MPP7	F: GCCTCCTATGCCTGATGACAT	81	NM_001100347.1
		R: CCCAGTGGTTCTCTATTTTTGACC		
	NELL2	F: AAGAGGGAGACGATGGACTGAG	105	NM_001102084.1
		R: ACACCAAGACCCCAAACTGCT		
	OSRS1*	F: GAGGATCTTGTGGAACCATTGA	124	FQ482089.2
		R: TACGGACAGCTGAACCCTTTC		
	OSRS2**	F: TTGTCAGTCCCCATCACCTTT	101	NW_003104406.1
		R: CATTGATTTGCACAGAAAACCA		
Downregulated genes	CD3G	F: AGCTTCAGACAAGCAGACGCT	101	BC103010.1
		R: GGGTTCAGTTCTTCCTCAGGTG		
	GNLY	F: TCCGTGCCAGTCAATCATGA	101	NM_001075143.1
		R: TGCAGACCTTGATGTCCACAC		
	LAPTM4B	F: GGTACTTGATCCTCAATGCCG	101	NM_205802.1
		R: AAAGTCACCCCCGAGTTCAGA		
	SRSF3	F: CGAAATGCATCGTGATTCCT	101	NM_001034700.1
		R: AATAGCCAAAAGCTCGTTCCA		

Primers (F: forward and R: reverse) used for gene amplification, amplicon size and GenBank accession numbers of the bovine cDNA sequences used for primer design.

*Ovine Scrapie Related Sequence 1 (OSRS1) has a high identity (92%) with the Bos taurus DNA sequence from clone CH240-103 G5(accession number: FQ482089.2) from 4172 to 4904 bp.

**Ovine Scrapie Related Sequence 2 (OSRS2) has a high identity (93%) with the Bos taurus chromosome 15 genomic scaffold, Bos_taurus_UMD_3.1, whole genome shotgun sequence (accession number: NW_003104406.1) from 2672152 to 2672419 bp.

qRT-PCR was performed using SYBR® Green (PE Applied Biosystems) assays. PCR amplification was performed in an ABI-Prism fast 7500 Sequence Detection System (PE Applied Biosystems). All qRT-PCR reactions were run in triplicate in total reaction volumes of 10 μl with 10–20 ng of cDNA as the template and a 300 nM final primer concentration. Universal conditions were used with an initial 10 min activation and denaturation step at 95°C, followed by 40 cycles of 15 s at 95°C and 30 s at 60°C. The baseline and threshold for the Ct calculations were set automatically with the ABI-Prism 7500 software Version 2.0.1. The levels of gene expression were determined using the comparative Ct method.

To improve the normalization accuracy, the geometric mean of three housekeeping genes was used to calculate the normalization factor (NF), which was used to normalize the expression level of each gene in each sample [37]. The NF was calculated from the GAPDH, G6PDH and RPL32 expression data. These are the three most stable reference housekeeping genes in the sheep medulla oblongata, and they have been used as internal references for expression studies in scrapie [38]. The primers and PCR conditions for the amplification of these housekeeping genes have been described previously [38,39].

The quantitative results obtained from the qRT-PCR assays were expressed as the fold-change. Student’s *t* test analyses were used to determine if the differences observed between the groups were statistically significant (*P* < 0.05).

## Results

### Preclinical scrapie-related lesions

**Figure 1:**
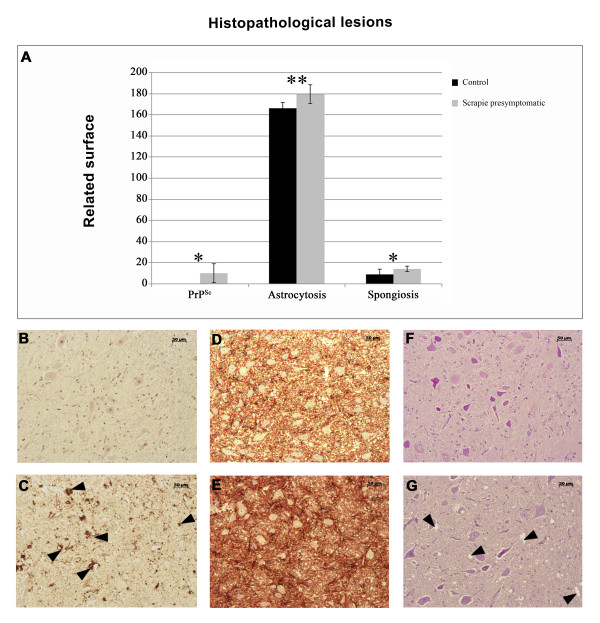
**Quantification of PrP**^**Sc**^**deposition, glial fibrillary acidic protein expression and spongiform degeneration.** The values represent the means (± standard deviation) of intensity of the DAB color (PrP^Sc^ and astrocytosis) and Haematoxiline & Eosine (spongiosis) obtained from ImageJ software (**A**). Grey bar correspond to scrapie-affected sheep and black bar to control sheep. Significant differences were detected using Student´s *t* test (**P < 0.01, *P < 0.05). A generalized increase in the expression of the astroglial marker glial fibrillary acidic protein (GFAP) was observed in the brains of the scrapie-affected sheep (*P* < 0.01). Hyperplasia and hypertrophy of the stellate GFAP-positive cells were observed in the medulla oblongata of the affected sheep, which is consistent with reactive astrocytosis. PrP^Sc^ staining in control (B) and scrapie medulla oblongata sample (**C**). GFAP staining in control (**D**) and scrapie medulla oblongata sample (**E**). Haematoxylin/Eosin staining in control (**F**) and scrapie medulla oblongata sample (**G**).

The neuropathological features of scrapie were evaluated in the medulla oblongata tissues of 6 control and 4 preclinical scrapie-infected sheep. Spongiosis, PrP^Sc^ deposition and GFAP immunoreactivity were consistent with the features of classical scrapie [40]. PrP^Sc^ deposition and spongiosis were only detected in the affected animals (Figure 1). Particular medullary areas in the obex, such as the nucleus dorsal motor of the vagus, the spinal tract of the trigeminal nerve and the solitary tract nucleus, were severely affected in the infected group. Even with the high variability observed in the scrapie group, the differences between the groups were statistically significant (*P* < 0.01 and *P* < 0.05). 

### Identification of the genes in the medulla oblongata that are differentially expressed in natural scrapie

**Figure 2:**
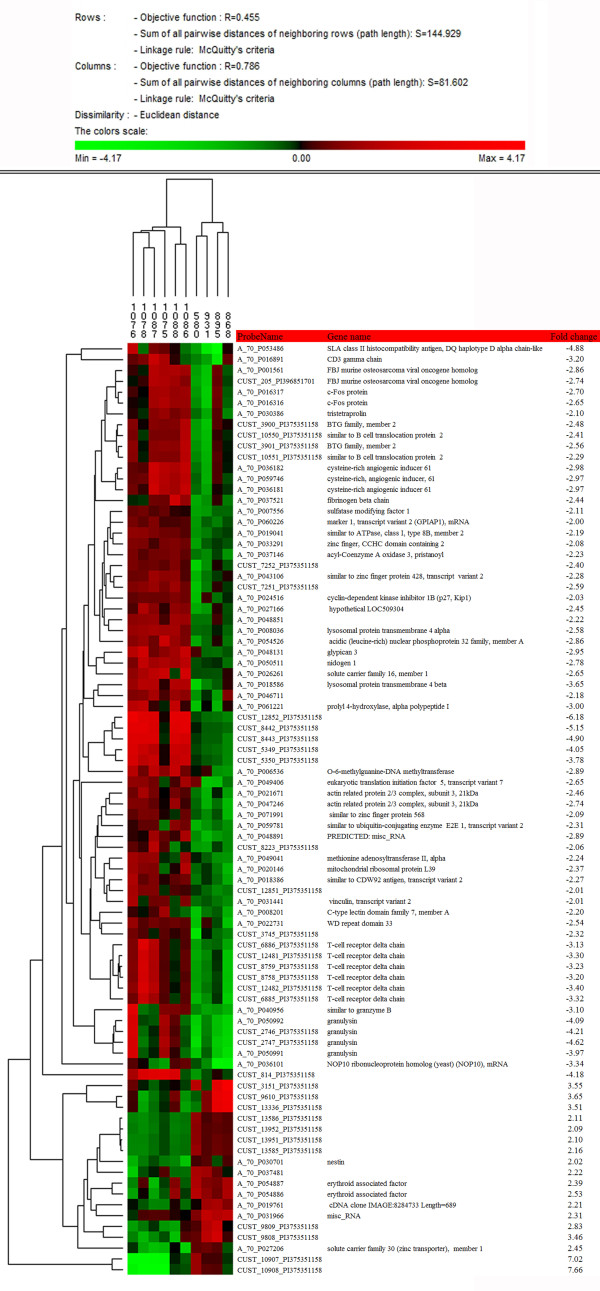
**Condition trees of the clustering analysis.** The hierarchical cluster analysis (Euclidean distance clustering algorithm) was performed using PermutMatrix [32], and 86 clones/genes differed significantly. Each colored bar represents a gene. The color represents the level of expression, and the sample information is listed across the top. The names of the known genes are indicated. Note the distinct patterns of altered gene expression between the positive and control animals.

A total of 86 probe sets displayed statistically significant differences between the control and scrapie preclinical groups that were equal to or greater than a 2-fold change. The genes from *Ovis aries* are relatively poorly annotated, but BLAST searches against publicly available databases allowed the identification of a set of 44 known genes from the complete set of 86 differentially expressed genes. The microarray data were deposited in the array express and are accessible through accession number E-MTAB-866. To determine the gene ontology (GO) categories of the deregulated genes in scrapie, we used DAVID Bioinformatics Resources 2008 [33,34] (NIAID/NIH, USA). Based on the GO analysis, 35 genes had known functions (Table 2), of which 3 were upregulated (5.7%) and 32 were downregulated (94.3%). The functional group with the highest number of regulated genes (18) was that of the phosphoprotein-encoding genes (Table 2). In addition, downregulated genes were included in GO groups encoding for proteins located in the lumen of organelles or the extracellular matrix and involved in the immune response and apoptosis. After the clustering analysis, the animals were grouped according to their disease condition (Figure 2). 

**Table 2:** Identified genes with known GO terms with a P ≤ 0.05 and ≥ 2-fold changes

					**Preclinical stage**	Clinical stage**
**Term**	**P-Value**	**Probe Name**	**Gene symbol**	**Gene name**	**FC**	
Compositionally biased region	**4.90E-02**	A_70_P049406	EIF5	Eukaryotic translation initiation factor 5, transcript variant 7	−2.65	−1.94
External side of plasma membrane	**4.60E-02**	A_70_P048131	GPC3	Glypican 3	−2.95	−2.56
Extracellular matrix binding	**5.30E-02**	A_70_P050511	NID1	Nidogen 1	−2.78	−4.15
Extracellular matrix organization	**1.90E-02**	A_70_P059746	CYR61	Cysteine-rich, angiogenic inducer, 61	−2.97	−3.42
		A_70_P061221	P4HA1	Prolyl 4-hydroxylase, alpha polypeptide I	−3.00	−2.81
Granzyme A mediated Apoptosis Pathway	**6.20E-02**	A_70_P054526	ANP32A	Acidic (leucine-rich) nuclear phosphoprotein 32 family, member A	−2.86	−2.47
		A_70_P040956	GZMB	Granzyme B	−3.10	NSR
MHC protein binding	**4.90E-02**	A_70_P016891	CD3G*	CD3 Gamma chain	−3.20	NSR
		A_70_P008201	CLEC7A	C-type lectin domain family 7, member A	−2.20	NSR
		CUST_12481_PI375351158	TRD@	T-cell receptor delta chain	−3.30	NSR
Organelle lumen	**6.80E-02**	A_70_P037521	FGB	Fibrinogen beta chain	−2.44	NSR
		A_70_P020146	MRPL39	Mitochondrial ribosomal protein L39	−2.37	NSR
		A_70_P036101	NOP10	NOP10 ribonucleoprotein homolog (yeast) (NOP10), mRNA	−3.34	NSR
		A_70_P007556	SUMF1	Sulfatase modifying factor 1	−2.11	NSR
Phosphoprotein	**1.30E-03**	A_70_P037146	ACOX3	Acyl-Coenzyme A oxidase 3, pristanoyl	−2.23	−1.73
		A_70_P021671	ARPC3	Actin related protein 2/3 complex, subunit 3, 21 kDa	−2.46	NSR
		A_70_P019041	ATP8B2	Similar to ATPase, class I, type 8B, member 2	−2.19	−1.78
		A_70_P024516	CDKN1B	Cyclin-dependent kinase inhibitor 1B (p27, Kip1)	−2.03	−1.71
		A_70_P001561	FOS	FBJ murine osteosarcoma viral oncogene homolog	−2.86	−2.66
		A_70_P008036	LAPTM4A	Lysosomal protein transmembrane 4 alpha	−2.58	−2.10
		A_70_P018586	LAPTM4B*	Lysosomal protein transmembrane 4 beta	−3.65	−2.80
		A_70_P049041	MAT2A	Methionine adenosyl transferase II, alpha	−2.24	−1.63
		A_70_P006536	MGMT	PREDICTED: O-6-methylguanine-DNA methyltransferase	−2.89	−2.22
		A_70_P030701	NES	Nestin	2.02	2.55
		A_70_P026261	SLC16A1	Solute carrier family 16, member 1 (monocarboxylic acid transporter 1)	−2.65	−2.34
		A_70_P027206	SLC30A1	Solute carrier family 30 (zinc transporter), member 1	2.45	2.27
		A_70_P018386	SLC44A1	CDW92 antigen, transcript variant 2	−2.27	NSR
		A_70_P059781	UBE2E1	Ubiquitin-conjugating enzyme E2E 1, transcript variant 2	−2.31	NSR
		A_70_P031441	VCL	Vinculin, transcript variant 2	−2.01	NSR
		A_70_P022731	WDR33	WD repeat domain 33	−2.54	NSR
		A_70_P033291	LOC618584	Zinc finger, CCHC domain containing 2	−2.08	−1.87
		A_70_P043106	ZNF428	Zinc finger protein 428, transcript variant 2	−2.28	−2.27
Response to a biotic stimulus	**4.00E-02**	CUST_10550_PI375351158	BTG2	B cell translocation protein 2	−2.41	NSR
Response to fungus	**4.90E-02**	A_70_P050991	GNLY*	Granulysin	−3.97	NSR
Tight junction assembly	**5.60E-02**	A_70_P003466	MPP7	membrane protein, palmitoylated 7 (MAGUK p55 subfamily member 7) (MPP7), mRNA	2.93	NSR

Shown are the Blast results of the clones with significant alterations in gene expression (P ≤ 0.05 and ≥ 2-fold change). FC of significant alteration during clinical stage is given in the last column (NSR: no significant regulation). The GO was determined using on-line functional annotation of DAVID Bioinformatics Resources 2008 (NIAID/NIH, USA). Only the genes with a known GO are shown.

*Genes chosen for validation by quantitative RT-PCR.

**FC values obtained in a previous study developed by our group comparing scrapie-infected animals in a clinical stage and controls [25].

### Identification of neuropathology-related genes

**Figure 3:**
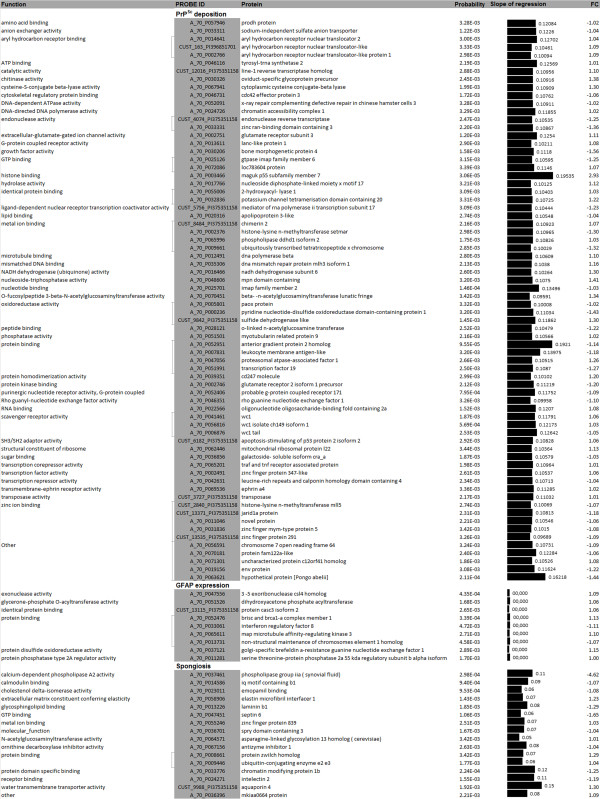
**Relationship between gene expression profiles and scrapie histopathological lesions.** Proteins encoded by genes whose expression is associated with PrP^Sc^ deposition, glial fibrillary acidic protein expression and spongiosis. Only the highly significant related genes are shown (P < 3.5x10^-3^). The slope of regression between histopathological lesions and gene expression was obtained under a Mixed Model approach.

We identified many genes with known functions whose expression was related to PrP^Sc^ deposition (1,011), although few genes were related to astrocytosis (21) and spongiosis (66). The expression of the genes that displayed a high probability of regression with scrapie lesions changed less than 2-fold (Figure 3). The gene ontology analysis revealed that genes associated with prion deposition encoded for proteins involved in protein and ion binding, oxidoreductase activity and transcription. Genes encoding for proteins involved in metal ion binding showed a positive association with both GFAP and spongiosis. In addition, genes encoding for proteins with oxidoreductase and phosphatase activity were associated with GFAP expression, and genes coding for extracellular matrix components or transmembrane transporters were associated with spongiosis. A list of known genes whose expression was highly correlated with PrP^Sc^ deposition, GFAP expression and spongiosis is shown in Figure 3. Only genes with a high probability of a positive (or negative) slope of regression are presented (P < 3.5x10^-3^).

### Validation of gene expression profiling by quantitative RT-PCR

To confirm the results of the microarray, we performed qRT-PCR using SYBR Green on a selected number of targets. For validation, we chose 4 genes from the expression study and 6 genes and 2 sequences from the association analysis. Eight of the genes/sequences were upregulated in the microarray (*APP*, *AQP4*, *CPPED1*, *GOLGA4*, *MPP7*, *NELL2*, OSRS1 and OSRS2) and four displayed downregulation (*CD3G*, *GNLY*, *LAPTM4B* and *SRSF3*). In most cases, the selection of genes was based on previous reports showing their associations with prion-related and other neurodegenerative diseases [41-44] but was also due to their potential involvement in the mechanisms involved in neurodegeneration.

**Figure 4:**
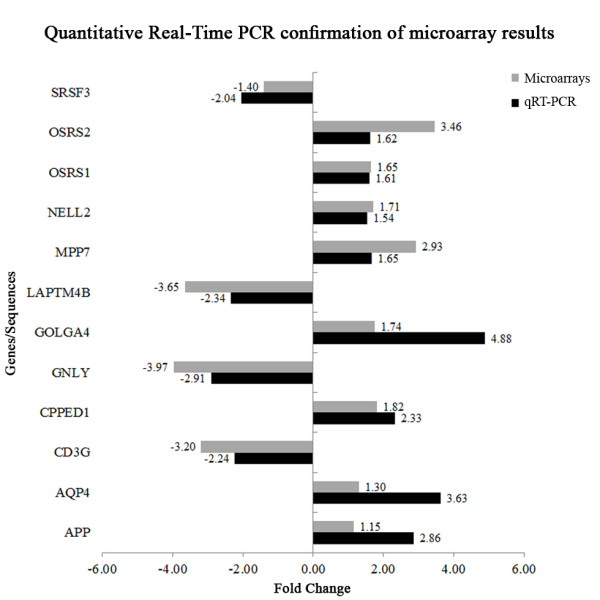
**Real-time RT-PCR confirmation of the microarray results.** Differential expression of selected sequences/genes analyzed by microarray and quantitative RT-PCR: amyloid beta (A4) precursor *(APP)*, aquaporin 4 *(AQP4)*, CD3 gamma chain *(CD3G)*, calcineurin-like phosphoesterase domain-containing 1 *(CPPED1)*, granulysin *(GNLY)*, golgi golgin subfamily 4 *(GOLGA4)*, lysosomal protein transmembrane 4 beta *(LAPTM4B)*, maguk p55 subfamily member 7 *(MPP7)*, nell2 *(NELL2)*, ovine scrapie related sequence 1 (OSRS1), ovine scrapie related sequence 2 (OSRS2) and serine/arginine-rich splicing factor 3 *(SRSF3)*.

The qRT-PCR analyses confirmed the microarray expression results (Figure 4). The differences between the control and scrapie groups were statistically significant for each of the 12 genes analyzed (P < 0.05).

## Discussion

Transmissible spongiform encephalopathies, or prion diseases, are fatal neurodegenerative diseases with characteristic spongiform lesions, neuronal cell loss, astrocytosis and the accumulation of the pathological form of the prion protein [45]. The precise mechanisms regulating these processes remain unknown. Genomic approaches are a potential tool to understand the molecular basis of complex mechanisms; in addition, they allow the discovery of new disease biomarkers.

The analysis of gene expression profiling can elucidate the molecular basis of this pathology. Several studies have focused on genomic analyses of brain tissue from animal models of prion diseases, including CJD, scrapie and BSE [19-24]. However, there are fewer studies involving the mRNA profiles of “natural” human CJD [46], bovine BSE [47] or ovine scrapie [48]. We previously reported a genomic analysis performed in tissues obtained from sheep naturally infected with scrapie in terminal stages [25]. However, the molecular changes can occur before the onset of the disease; for example, PrP^Sc^ accumulation occurs early in the disease in both the central nervous system [49] and peripheral tissues [50]. In mouse models, genomic expression profiles revealed the induction of oxidative and endoplasmic reticulum (ER) stress, activated ER and mitochondrial apoptosis pathways, and activated cholesterol biosynthesis in the central nervous system of preclinical mice [17]. We report here the first transcriptome study of the central nervous system (CNS) in sheep naturally infected with scrapie in preclinal stages that associated the variations in the expression profile with the features of scrapie neuropathology.

### Differential gene expression in preclinical scrapie

Our microarray hybridization analysis identified 86 probes with changes in expression greater than 2-fold compared with the controls. Using the same platform, our previous study of sheep clinically affected with scrapie [25] revealed 350 differentially regulated probes, indicating that at the early stages of the disease, fewer genes are active or the expression changes are not high enough to be detected by microarray. From the 86 probes, 40 were also differentially regulated in clinical animals [25] and 46 were identified only in preclinical sheep (some of these probes are shown in Table 2).

In vitro studies have shown variations in the phospho-proteome of N2a cells after PrP^Sc^ infection [51] and specific inflammatory profiles in microglia [52]. In addition, genes involved in defense, the immune response or encoding for secreted extracellular proteins are differentially regulated in murine models during prion infection [20]. In the clinical phase of classical scrapie, the genes involved in ion-binding and transport, nucleotide binding and the immune system are differentially expressed [25]. In accordance with the in vitro and murine models and with our previous results in classical scrapie at the late stages of the disease, the differentially regulated genes identified in this study encoded phospho-proteins, extracellular matrix organization proteins and immune response-related proteins. Therefore, these mechanisms seem to be involved in the neuropathology of scrapie from the early phases of the disease.

The microarray analysis was validated using qRT-PCR for 4 genes; their differential regulation was confirmed in all cases. The high adjustment between the fold changes obtained with the microarray and the qRT-PCR of the genes selected for confirmation reflects the high credibility of the microarray and the gene alignment analysis. Three of these genes were downregulated in scrapie tissues. Two of them, CD3G and GNLY, are involved in the immune response. CD3G is part of the T-cell receptor-CD3 complex, which plays an important role in coupling antigen recognition to several intracellular signal-transduction pathways [53]. The CD3 T cells accumulate near or around blood vessels and in the CNS parenchyma of mice inoculated with scrapie [54], suggesting the infiltration of T cells in the brain. In contrast, the downregulation of *CD3G* observed in our natural model suggests that these cells are decreased in preclinical scrapie. Similarly, we observed a significant decrease in the expression of *GNLY*, which is a potent antimicrobial protein contained within the granules of CTL and NK cells [55]. Taking together, our results suggest a decline of immune activity in prion diseases, as described for other neurodegenerative diseases such as Alzheimer’s disease (AD) [56].

Another gene that was downregulated in the preclinical medullae encodes the lysosomal protein transmembrane 4 protein (LAPTM4). The endosomal and lysosomal compartments are implicated in trafficking, recycling and the final degradation of prions [57]. It has been proposed that autophagy might play a protective role in prion diseases, leading to the degradation of prions. Galectin-3 knockout mice express low levels of lysosomal activation marker (LAMP-2) and autophagy markers, suggesting that endosomal/lysosomal dysfunction in combination with reduced autophagy may contribute to the development of prion diseases [58]. The downregulation of LAPTM4 is in accordance with these results and might indicate a dysfunction of the lysosomal-endosomal pathway in preclinical scrapie.

One of the genes upregulated in the microarray hybridization analysis was Maguk p55 subfamily member 7 *MPP7*. The membrane-associated guanylate kinase homologues (MAGUKs) are a family of peripheral membrane proteins that form multiprotein complexes containing distinct sets of transmembrane, cytoskeletal, and cytoplasmic signaling proteins. MPP7 acts as an important adapter that promotes epithelial cell polarity, tight junction formation via its interaction with DLG1 and is involved in the assembly of protein complexes at sites of cell-cell contact [59]. The cellular prion protein PrP^c^ is also located at cell-cell adhesion sites in polarized/differentiated enterocytes and interacts with desmosomal proteins and with actin and actin-binding proteins at cell-cell junctions [60]. Moreover, in the CNS, the PrP^c^ is located in the microvascular endothelium and at intercellular junctions of cultured brain endothelial cells of mouse, rat and human origin [61]. We report here for the first time the upregulation of the gene encoding the MPP7 protein in preclinical scrapie and its positive association with PrP^Sc^ deposition, suggesting a possible alteration of cell-cell adhesion the early stages of the disease.

### The genomic association with scrapie-related lesions

Studies of the associations between gene expression and the intensity of scrapie lesions have been shown to be a powerful tool to detect genes potentially involved in the development of these lesions [25]. In the present study, we found a relatively low number of genes with differential expression in the preclinical tissues; however, the association study allowed the identification of genes that had slight changes (FC < 2) in the microarray hybridization analysis but whose expression was strongly related to the development of the scrapie-related lesions. The genes that displayed a positive regression with prion deposition were involved in several cellular mechanisms, the most frequent of which being protein, metal and ion binding, oxidoreductase activity and transcription factors. The possible role of PrP^c^ to protect cells from oxidative stress is well documented [62], as is the capacity of the prion protein to bind Cu^2+^[63,64]. In addition, many other roles have been attributed to the prion protein, such as transmembrane signaling or cell adhesion [65]. Genes involved in these mechanisms have been shown to be associated with prion diseases, astrocytosis or spongiosis, thereby corroborating the reliability of our association study.

The expression of 6 genes and 2 sequences was validated using qRT-PCR. These genes were chosen because of their known role in brain metabolism and/or neurodegeneration, and the sequences displayed the most significant degrees of association with scrapie lesions. We found a slight up-regulation of the amyloid beta (A4) precursor protein (APP). APP and PrP are both cell-surface proteins residing in cholesterol-rich lipid rafts of the cell membrane and play an important role in the development of AD and prion diseases, respectively. These diseases share a number of clinical, pathological and biochemical characteristics [66]. The brains of CJD patients display the pathology of both prion diseases and AD [67]. The overexpression of *APP* in other genetic expression profiling studies in scrapie murine models has been previously reported [24]. In addition, the overexpression of *APP* facilitates the rapid development of artificial scrapie [68]. The overexpression of *APP* in the preclinical naturally infected animals found in our study are in accordance with these previous studies and highlight the possible early interaction between APP and PrP.

Water metabolism is of major importance in a number of physiological processes in the CNS. Alterations in the distribution of water and cerebrospinal fluid in the brain are a common occurrence in multiple neuropathological conditions, including brain edema, brain tumors, stroke, hyponatremia, head injuries and hydrocephalus. Aquaporin 4 (*AQP4*) is most likely expressed by activated glial cells, and an increase in its level is indicative of ongoing astrocytosis [69]. The increase in the expression levels of *AQP4* has been reported in Creutzfeldt-Jakob disease, bovine spongiform encephalopathy and scrapie-infected transgenic mice [22,43,44,70]. Our work confirms the upregulation of *AQP4* in the preclinical phases of natural ovine scrapie.

Genes that have never been associated with prion diseases or other neurodegenerative diseases were shown to be significantly regulated. Our microarray data indicated an increase in the expression of a gene similar to calcineurin-like phosphoesterase domain-containing 1 *CPPED1* that was confirmed by quantitative RT-PCR. The CPPED1 protein has hydrolase and metal ion-binding activities. To date, no studies have reported the differential regulation of this gene in neurodegenerative diseases. However, PrP^c^ interacts with a range of divalent metal ions and maintains the their homeostasis, and the conformational change that occurs in the formation of PrP^Sc^ is induced by the interaction with ions (see [71] for review). This gene has a positive association with prion deposition (GEAMM analysis), suggesting a possible role in early scrapie development. However, further analysis will be essential to confirm this conclusion.

Golgi golgin subfamily 4 *GOLGA4* may play a role in the delivery of transport vesicles containing GPI-linked proteins from the trans-Golgi network through its interaction with microtubule-actin crosslinking factor 1 (MACF1). The prion protein is attached to the outer leaflet of the plasma membrane by a glycosyl-phosphatidyl-inositol (GPI) anchor (reviewed in [72]). Our results demonstrate the upregulation of *GOLGA4* is positively associated with PrP^Sc^ deposition, suggesting that this protein might have a role in PrP trafficking.

We previously reported that anti-apoptotic genes are overexpressed in terminal scrapie, which suggested the activation of neuroprotective mechanisms during the disease [30]. In accordance with this, we found here that neural tissue-specific epidermal growth factor-like repeat domain-containing protein) NELL2 is overexpressed in preclinical scrapie. NELL2 is a secreted glycoprotein that is predominantly expressed in neural tissues and increases *in vitro* cell survival under cell death-inducing conditions [73]. In addition, NELL2 may play an important role in the maintenance of neural functions by regulating the intracellular machinery involving Ca^2+^ signaling, synaptic transport and/or vesicle release [74]. In our study, NELL2 displayed a positive association with PrP^Sc^ deposition and spongiosis, which suggests a possible role in the pathogenesis of the disease related to the role of PrP in Ca^2+^ homeostasis [75].

Finally, we observed the downregulation of a serine/arginine-rich splicing factor 3 *SRSF3*, which seems to be involved in the differential splicing of the low-density lipoprotein receptor (LDLR), a major apolipoprotein E (APOE) receptor that has been associated with cholesterol homeostasis and, possibly, AD development [76]. This splicing factor is a proto-oncogene [77] and is antiapoptotic [78]. To our knowledge, our work is the first study to describe the differential regulation of this gene in prion diseases. The downregulation of *SRSF3* is in accordance with its probable protective activity against neuronal cell death. Further studies will be necessary to investigate the possible role of SRSF3 in the disease.

In addition to the regulation of known genes, several non-annotated sequences were differentially expressed in the preclinical medullae and associated with scrapie lesions. We confirmed the upregulation of two sequences (OSRS1 and OSRS2) that were associated with astrocytosis and spongiosis, respectively. These sequences did not display homology with any known genes (Table 1), but they show a high homology with parts of two published bovine sequences (FQ482089.2 and NW_003104406.1, respectively). These sequences come from an ovine cDNA library generated from the brain and lymphoid tissue of scrapie- and control-infected sheep [25]. Although further analyses are necessary to confirm their differential regulation in a wider number of animals or in different prion animal models, these custom sequences can represent potential unknown biomarkers useful for the diagnosis of presymptomatic prion disease.

## Conclusions

In summary, this is the first genome-wide expression study performed in naturally infected sheep with preclinical scrapie and shows the induction of a reduced number of genes compared with the changes shown in clinical scrapie sheep. Differentially regulated genes confirmed the involvement of the immune system, alterations in the extracellular matrix and changes in the ion binding in the neuropathology of prion diseases. In addition, changes in the levels of genes encoding for proteins related to cell-cell contact and trafficking and re-cycling pathways could play an important role in the development of the disease. The association of genomic changes with scrapie lesions allowed the identification of a higher number of candidate genes to be used as biomarkers and could be useful to develop biotools for the early diagnosis of the disease. In addition to their inclusion in the previous functional groups, the identified genes were related to water metabolism or apoptosis. As in previous studies, our findings confirm the close relationship between scrapie and other neurodegenerative diseases. Moreover, the reported association analysis contributes to the knowledge of the molecular mechanisms underlying the pathogenesis of prion diseases. Further studies are required to locate the cellular proteins encoded by these differentially regulated genes and to study the expression of the identified genes in other brain areas and, in this manner, contribute to the knowledge of their role in the disease.

## Abbreviations

APP: Amyloid beta (A4) precursor; AQP4: Aquaporin 4; ARQ: Alanine arginine glutamine; BSE: Bovine spongiform encephalopathy; CD3G: Cluster of differentiation 3 gamma chain; CPPED1: Calcineurin-like phosphoesterase domain-containing 1; DAB: 3,3'-diaminobenzidine; G6PDH: Glucose-6-phosphate dehydrogenase; GAPDH: Glyceraldehyde-3-phosphate dehydrogenase; GEAMM: Gene expression analysis with mixed models; GNLY: Granulysin; GO: Gene ontology; GOLGA4: Golgi golgin subfamily 4; HE: Hematoxylin eosine; IHC: Immunohistochemistry; LAPTM4B: Lysosomal protein transmembrane 4 beta; MO: Medulla oblongata; MPP7: Maguk p55 subfamily member 7; OSRS: Ovine scrapie related sequences; PRNP: Prion protein; PrP^C^: Cellular prion protein; PrP^Sc^: Scrapie prion protein; RIN: RNA integrity number; RPL32: Ribosomal protein l32; SRSF3: Serine/arginine-rich splicing factor 3; TSEs: Transmissible spongiform encephalopathies; VRQ: Valine arginine glutamine.

## Competing interests

The authors declare that they have no competing interests.

## Authors’ contributions

HF performed the experiments and drafted the manuscript. IMB participated in the design of the study, participated in the molecular genetic studies, in the sequence alignment and drafted the manuscript. FH participated in carrying out the microarray analysis. LV performed the association analysis. CS participated in carrying out of the genetic studies. CA participated in the design of the study and drafted the manuscript. JJB participated in its design and coordination and helped to draft the manuscript. AB participated in the design of the microarray study and sequence alignment. RB conceived of the study, and participated in its design and coordination and helped to draft the manuscript. All authors read and approved the final manuscript.

## References

[B1] Prusiner SB Prions Proc Natl Acad Sci U S A 1998 95 13363 13383 10.1073/pnas.95.23.13363 9811807 PMC33918

[B2] Griffith JS Self-replication and scrapie Nature 1967 215 1043 1044 10.1038/2151043a0 4964084

[B3] Prusiner SB Novel proteinaceous infectious particles cause scrapie Science 1982 216 136 144 10.1126/science.6801762 6801762

[B4] Aguzzi A Weissmann C Brandner S Raeber AJ Klein MA Voigtlander T PrP-expressing tissue required for transfer of scrapie infectivity from spleen to brain Nature 1997 389 69 73 10.1038/37981 9288968

[B5] Chesebro B Human TSE disease–viral or protein only? Nat Med 1997 3 491 492 10.1038/nm0597-491 9142109

[B6] Bons N Mestre-Frances N Belli P Cathala F Gajdusek DC Brown P Natural and experimental oral infection of nonhuman primates by bovine spongiform encephalopathy agents Proc Natl Acad Sci U S A 1999 96 4046 4051 10.1073/pnas.96.7.4046 10097160 PMC22417

[B7] Andreoletti O Berthon P Marc D Sarradin P Grosclaude J van Keulen L Schelcher F Elsen J Lantier F Early accumulation of PrPsc in gut-associated lymphoid and nervous tissues of susceptible sheep from a Romanov flock with natural scrapie J Gen Virol 2000 81 3115 3126 11086143 10.1099/0022-1317-81-12-3115

[B8] van Keulen LJ Schreuder BE Vromans ME Langeveld JP Smits MA Pathogenesis of natural scrapie in sheep Arch Virol Suppl 2000 16 57 71 11214935 10.1007/978-3-7091-6308-5_5

[B9] van Keulen LJ Bossers A van Zijderveld F TSE pathogenesis in cattle and sheep Vet Res 2008 39 24 10.1051/vetres:2007061 18258167

[B10] Elsen JM Amigues Y Schelcher F Ducrocq V Andreoletti O Eychenne F Khang JV Poivey JP Lantier F Laplanche JL Genetic susceptibility and transmission factors in scrapie: detailed analysis of an epidemic in a closed flock of Romanov Arch Virol 1999 144 431 445 10.1007/s007050050516 10226611

[B11] Diaz C Vitezica ZG Rupp R Andreoletti O Elsen JM Polygenic variation and transmission factors involved in the resistance/susceptibility to scrapie in a Romanov flock J Gen Virol 2005 86 849 857 10.1099/vir.0.80412-0 15722548

[B12] Aguzzi A Peripheral prion pursuit J Clin Invest 2001 108 661 662 11544269 10.1172/JCI13919 PMC209391

[B13] Collinge J Owen F Poulter M Leach M Crow TJ Rossor MN Hardy J Mullan MJ Janota I Lantos PL Prion dementia without characteristic pathology Lancet 1990 336 7 9 10.1016/0140-6736(90)91518-F 1973256

[B14] Manetto V Medori R Cortelli P Montagna P Tinuper P Baruzzi A Rancurel G Hauw JJ Vanderhaeghen JJ Mailleux P Fatal familial insomnia: clinical and pathologic study of five new cases Neurology 1992 42 312 319 10.1212/WNL.42.2.312 1736158

[B15] NC Mallucci GR Rescuing neurons in prion disease Biochem J 433 19 29 21158739 10.1042/BJ20101323

[B16] Yun SW Gerlach M Riederer P Klein MA Oxidative stress in the brain at early preclinical stages of mouse scrapie Exp Neurol 2006 201 90 98 10.1016/j.expneurol.2006.03.025 16806186

[B17] Brown AR Rebus S McKimmie CS Robertson K Williams A Fazakerley JK Gene expression profiling of the preclinical scrapie-infected hippocampus Biochem Biophys Res Commun 2005 334 86 95 10.1016/j.bbrc.2005.06.060 15992767

[B18] Tortosa R Castells X Vidal E Costa C Ruiz De Villa Mdel C Sanchez A Barcelo A Torres JM Pumarola M Arino J Central nervous system gene expression changes in a transgenic mouse model for bovine spongiform encephalopathy Vet Res 2011 42 109 10.1186/1297-9716-42-109 22035425 PMC3225326

[B19] Dandoy-Dron F Guillo F Benboudjema L Deslys JP Lasmezas C Dormont D Tovey MG Dron M Gene expression in scrapie. Cloning of a new scrapie-responsive gene and the identification of increased levels of seven other mRNA transcripts J Biol Chem 1998 273 7691 7697 10.1074/jbc.273.13.7691 9516475

[B20] Booth S Bowman C Baumgartner R Sorensen G Robertson C Coulthart M Phillipson C Somorjai RL Identification of central nervous system genes involved in the host response to the scrapie agent during preclinical and clinical infection J Gen Virol 2004 85 3459 3471 10.1099/vir.0.80110-0 15483264

[B21] Brown AR Webb J Rebus S Williams A Fazakerley JK Identification of up-regulated genes by array analysis in scrapie-infected mouse brains Neuropathol Appl Neurobiol 2004 30 555 567 10.1111/j.1365-2990.2004.00565.x 15488032

[B22] Riemer C Neidhold S Burwinkel M Schwarz A Schultz J Kratzschmar J Monning U Baier M Gene expression profiling of scrapie-infected brain tissue Biochem Biophys Res Commun 2004 323 556 564 10.1016/j.bbrc.2004.08.124 15369787

[B23] Xiang W Windl O Wunsch G Dugas M Kohlmann A Dierkes N Westner IM Kretzschmar HA Identification of differentially expressed genes in scrapie-infected mouse brains by using global gene expression technology J Virol 2004 78 11051 11060 10.1128/JVI.78.20.11051-11060.2004 15452225 PMC521804

[B24] Skinner PJ Abbassi H Chesebro B Race RE Reilly C Haase AT Gene expression alterations in brains of mice infected with three strains of scrapie BMC Genomics 2006 7 114 10.1186/1471-2164-7-114 16700923 PMC1475852

[B25] Filali H Martin-Burriel I Harders F Varona L Lyahyai J Zaragoza P Pumarola M Badiola JJ Bossers A Bolea R Gene expression profiling and association with prion-related lesions in the medulla oblongata of symptomatic natural scrapie animals PLoS One 2011 6 e19909 10.1371/journal.pone.0019909 21629698 PMC3101219

[B26] Vargas F Lujan L Bolea R Monleon E Martin-Burriel I Fernandez A De Blas I Badiola JJ Detection and clinical evolution of scrapie in sheep by 3rd eyelid biopsy J Vet Intern Med 2006 20 187 193 10.1111/j.1939-1676.2006.tb02840.x 16496940

[B27] Bolea R Monleon E Schiller I Raeber AJ Acin C Monzon M Martin-Burriel I Struckmeyer T Oesch B Badiola JJ Comparison of immunohistochemistry and two rapid tests for detection of abnormal prion protein in different brain regions of sheep with typical scrapie J Vet Diagn Invest 2005 17 467 469 10.1177/104063870501700511 16312240

[B28] Acin C Martin-Burriel I Monleon E Rodellar C Badiola JJ Zaragoza P PrP polymorphisms in Spanish sheep affected with natural scrapie Vet Rec 2004 155 370 372 10.1136/vr.155.12.370 15493607

[B29] Monleon E Monzon M Hortells P Vargas A Acin C Badiola JJ Detection of PrPsc on lymphoid tissues from naturally affected scrapie animals: comparison of three visualization systems J Histochem Cytochem 2004 52 145 151 10.1177/002215540405200201 14729865

[B30] Serrano C Lyahyai J Bolea R Varona L Monleon E Badiola JJ Zaragoza P Martin-Burriel I Distinct spatial activation of intrinsic and extrinsic apoptosis pathways in natural scrapie: association with prion-related lesions Vet Res 2009 40 42 10.1051/vetres/2009024 19401142 PMC2701179

[B31] Vidal E Acin C Foradada L Monzon M Marquez M Monleon E Pumarola M Badiola JJ Bolea R Immunohistochemical characterisation of classical scrapie neuropathology in sheep J Comp Pathol 2009 141 135 146 10.1016/j.jcpa.2009.04.002 19515381

[B32] Caraux G Pinloche S PermutMatrix: a graphical environment to arrange gene expression profiles in optimal linear order Bioinformatics 2005 21 1280 1281 10.1093/bioinformatics/bti141 15546938

[B33] Dennis G Jr Sherman BT Hosack DA Yang J Gao W Lane HC Lempicki RA DAVID Database for annotation, visualization, and integrated discovery Genome Biol 2003 4 P3 10.1186/gb-2003-4-5-p3 12734009 PMC193660

[B34] da Huang W Sherman BT Lempicki RA Systematic and integrative analysis of large gene lists using DAVID bioinformatics resources Nat Protoc 2009 4 44 57 19131956 10.1038/nprot.2008.211

[B35] Casellas J Ibanez-Escriche N Martinez-Giner M Varona L GEAMM v.1.4: a versatile program for mixed model analysis of gene expression data Anim Genet 2008 39 89 90 10.1111/j.1365-2052.2007.01670.x 18076745

[B36] Gelfand AE Smith AFM Sampling-Based Approaches to Calculating Marginal Densities J Am Stat Assoc 1990 85 398 409 10.1080/01621459.1990.10476213

[B37] Vandesompele J De Preter K Pattyn F Poppe B Van Roy N De Paepe A Speleman F Accurate normalization of real-time quantitative RT-PCR data by geometric averaging of multiple internal control genes Genome Biol 2002 3 RESEARCH0034 12184808 10.1186/gb-2002-3-7-research0034 PMC126239

[B38] Lyahyai J Serrano C Ranera B Badiola JJ Zaragoza P Martin-Burriel I Effect of scrapie on the stability of housekeeping genes Anim Biotechnol 2010 21 1 13 20024782 10.1080/10495390903323851

[B39] Garcia-Crespo D Juste RA Hurtado A Selection of ovine housekeeping genes for normalisation by real-time RT-PCR; analysis of PrP gene expression and genetic susceptibility to scrapie BMC Vet Res 2005 1 3 10.1186/1746-6148-1-3 16188044 PMC1262732

[B40] Vidal E Bolea R Tortosa R Costa C Domenech A Monleon E Vargas A Badiola JJ Pumarola M Assessment of calcium-binding proteins (Parvalbumin and Calbindin D-28 K) and perineuronal nets in normal and scrapie-affected adult sheep brains J Virol Methods 2006 136 137 146 10.1016/j.jviromet.2006.05.008 16828173

[B41] Endres K Mitteregger G Kojro E Kretzschmar H Fahrenholz F Influence of ADAM10 on prion protein processing and scrapie infectiosity in vivo Neurobiol Dis 2009 36 233 241 10.1016/j.nbd.2009.07.015 19632330

[B42] Parkin ET Watt NT Hussain I Eckman EA Eckman CB Manson JC Baybutt HN Turner AJ Hooper NM Cellular prion protein regulates beta-secretase cleavage of the Alzheimer's amyloid precursor protein Proc Natl Acad Sci U S A 2007 104 11062 11067 10.1073/pnas.0609621104 17573534 PMC1904148

[B43] Costa C Tortosa R Rodriguez A Ferrer I Torres JM Bassols A Pumarola M Aquaporin 1 and aquaporin 4 overexpression in bovine spongiform encephalopathy in a transgenic murine model and in cattle field cases Brain Res 2007 1175 96 106 17868659 10.1016/j.brainres.2007.06.088

[B44] Rodriguez A Perez-Gracia E Espinosa JC Pumarola M Torres JM Ferrer I Increased expression of water channel aquaporin 1 and aquaporin 4 in Creutzfeldt-Jakob disease and in bovine spongiform encephalopathy-infected bovine-PrP transgenic mice Acta Neuropathol 2006 112 573 585 10.1007/s00401-006-0117-1 16871401

[B45] Prusiner SB Bolton DC Groth DF Bowman KA Cochran SP McKinley MP Further purification and characterization of scrapie prions Biochemistry 1982 21 6942 6950 10.1021/bi00269a050 6818988

[B46] Xiang W Windl O Westner IM Neumann M Zerr I Lederer RM Kretzschmar HA Cerebral gene expression profiles in sporadic Creutzfeldt-Jakob disease Ann Neurol 2005 58 242 257 10.1002/ana.20551 16049922

[B47] Khaniya B Almeida L Basu U Taniguchi M Williams JL Barreda DR Moore SS Guan LL Microarray analysis of differentially expressed genes from Peyer's patches of cattle orally challenged with bovine spongiform encephalopathy J Toxicol Environ Health A 2009 72 1008 1013 10.1080/15287390903084199 19697233

[B48] Cosseddu GM Andreoletti O Maestrale C Robert B Ligios C Piumi F Agrimi U Vaiman D Gene expression profiling on sheep brain reveals differential transcripts in scrapie-affected/not-affected animals Brain Res 2007 1142 217 222 17303092 10.1016/j.brainres.2007.01.033

[B49] Andreoletti O Simon S Lacroux C Morel N Tabouret G Chabert A Lugan S Corbiere F Ferre P Foucras G PrPSc accumulation in myocytes from sheep incubating natural scrapie Nat Med 2004 10 591 593 10.1038/nm1055 15156203

[B50] Casalone C Corona C Crescio MI Martucci F Mazza M Ru G Bozzetta E Acutis PL Caramelli M Pathological prion protein in the tongues of sheep infected with naturally occurring scrapie J Virol 2005 79 5847 5849 10.1128/JVI.79.9.5847-5849.2005 15827199 PMC1082725

[B51] Wagner W Ajuh P Lower J Wessler S Quantitative phosphoproteomic analysis of prion-infected neuronal cells Cell Commun Signal 2010 8 28 10.1186/1478-811X-8-28 20920157 PMC2955621

[B52] Baker CA Manuelidis L Unique inflammatory RNA profiles of microglia in Creutzfeldt-Jakob disease Proc Natl Acad Sci U S A 2003 100 675 679 10.1073/pnas.0237313100 12525699 PMC141055

[B53] Flanagan BF Wotton D Tuck-Wah S Owen MJ DNase hypersensitivity and methylation of the human CD3G and D genes during T-cell development Immunogenetics 1990 31 13 20 10.1007/BF00702484 2137107

[B54] Lewicki H Tishon A Homann D Mazarguil H Laval F Asensio VC Campbell IL DeArmond S Coon B Teng C T cells infiltrate the brain in murine and human transmissible spongiform encephalopathies J Virol 2003 77 3799 3808 10.1128/JVI.77.6.3799-3808.2003 12610154 PMC149501

[B55] Hogg AE Bowick GC Herzog NK Cloyd MW Endsley JJ Induction of granulysin in CD8+ T cells by IL-21 and IL-15 is suppressed by human immunodeficiency virus-1 J Leukoc Biol 2009 86 1191 1203 10.1189/jlb.0409222 19687290

[B56] Richartz-Salzburger E Batra A Stransky E Laske C Kohler N Bartels M Buchkremer G Schott K Altered lymphocyte distribution in Alzheimer's disease J Psychiatr Res 2007 41 174 178 10.1016/j.jpsychires.2006.01.010 16516234

[B57] Heiseke A Aguib Y Schatzl HM Autophagy, prion infection and their mutual interactions Curr Issues Mol Biol 2010 12 87 97 19767652

[B58] Mok SW Riemer C Madela K Hsu DK Liu FT Gultner S Heise I Baier M Role of galectin-3 in prion infections of the CNS Biochem Biophys Res Commun 2007 359 672 678 10.1016/j.bbrc.2007.05.163 17555713

[B59] Stucke VM Timmerman E Vandekerckhove J Gevaert K Hall A The MAGUK protein MPP7 binds to the polarity protein hDlg1 and facilitates epithelial tight junction formation Mol Biol Cell 2007 18 1744 1755 10.1091/mbc.E06-11-0980 17332497 PMC1855022

[B60] Morel E Fouquet S Strup-Perrot C Pichol Thievend C Petit C Loew D Faussat AM Yvernault L Pincon-Raymond M Chambaz J The cellular prion protein PrP(c) is involved in the proliferation of epithelial cells and in the distribution of junction-associated proteins PLoS One 2008 3 e3000 10.1371/journal.pone.0003000 18714380 PMC2500194

[B61] Viegas P Chaverot N Enslen H Perriere N Couraud PO Cazaubon S Junctional expression of the prion protein PrPC by brain endothelial cells: a role in trans-endothelial migration of human monocytes J Cell Sci 2006 119 4634 4643 10.1242/jcs.03222 17062642

[B62] Milhavet O Lehmann S Oxidative stress and the prion protein in transmissible spongiform encephalopathies Brain Res Brain Res Rev 2002 38 328 339 11890980 10.1016/s0165-0173(01)00150-3

[B63] Vassallo N Herms J Cellular prion protein function in copper homeostasis and redox signalling at the synapse J Neurochem 2003 86 538 544 10.1046/j.1471-4159.2003.01882.x 12859667

[B64] Millhauser GL Copper and the prion protein: methods, structures, function, and disease Annu Rev Phys Chem 2007 58 299 320 10.1146/annurev.physchem.58.032806.104657 17076634 PMC2904554

[B65] Westergard L Christensen HM Harris DA The cellular prion protein (PrP(C)): its physiological function and role in disease Biochim Biophys Acta 2007 1772 629 644 10.1016/j.bbadis.2007.02.011 17451912 PMC1986710

[B66] Barnham KJ Cappai R Beyreuther K Masters CL Hill AF Delineating common molecular mechanisms in Alzheimer's and prion diseases Trends Biochem Sci 2006 31 465 472 10.1016/j.tibs.2006.06.006 16820299

[B67] Hainfellner JA Wanschitz J Jellinger K Liberski PP Gullotta F Budka H Coexistence of Alzheimer-type neuropathology in Creutzfeldt-Jakob disease Acta Neuropathol 1998 96 116 122 10.1007/s004010050870 9705125

[B68] Baier M Apelt J Riemer C Gultner S Schwarz A Bamme T Burwinkel M Schliebs R Prion infection of mice transgenic for human APPSwe: increased accumulation of cortical formic acid extractable Abeta(1–42) and rapid scrapie disease development Int J Dev Neurosci 2008 26 821 824 10.1016/j.ijdevneu.2008.07.001 18662767

[B69] Nielsen S Nagelhus EA Amiry-Moghaddam M Bourque C Agre P Ottersen OP Specialized membrane domains for water transport in glial cells: high-resolution immunogold cytochemistry of aquaporin-4 in rat brain J Neurosci 1997 17 171 180 8987746 10.1523/JNEUROSCI.17-01-00171.1997 PMC6793699

[B70] Riemer C Queck I Simon D Kurth R Baier M Identification of upregulated genes in scrapie-infected brain tissue J Virol 2000 74 10245 10248 10.1128/JVI.74.21.10245-10248.2000 11024157 PMC102067

[B71] Rana A Gnaneswari D Bansal S Kundu B Prion metal interaction: is prion pathogenesis a cause or a consequence of metal imbalance? Chem Biol Interact 2009 181 282 291 10.1016/j.cbi.2009.07.021 19660443

[B72] Nunziante M Gilch S Schatzl HM Prion diseases: from molecular biology to intervention strategies ChemBioChem 2003 4 1268 1284 10.1002/cbic.200300704 14661267

[B73] Choi EJ Kim DH Kim JG Kim DY Kim JD Seol OJ Jeong CS Park JW Choi MY Kang SG Estrogen-dependent transcription of the NEL-like 2 (NELL2) gene and its role in protection from cell death J Biol Chem 2010 285 25074 25084 10.1074/jbc.M110.100545 20538601 PMC2915743

[B74] Kim H Ha CM Choi J Choi EJ Jeon J Kim C Park SK Kang SS Kim K Lee BJ Ontogeny and the possible function of a novel epidermal growth factor-like repeat domain-containing protein, NELL2, in the rat brain J Neurochem 2002 83 1389 1400 10.1046/j.1471-4159.2002.01245.x 12472893

[B75] Brini M Miuzzo M Pierobon N Negro A Sorgato MC The prion protein and its paralogue Doppel affect calcium signaling in Chinese hamster ovary cells Mol Biol Cell 2005 16 2799 2808 10.1091/mbc.E04-10-0915 15788568 PMC1142425

[B76] Ling IF Estus S Role of SFRS13A in low-density lipoprotein receptor splicing Hum Mutat 2010 31 702 709 10.1002/humu.21244 20232416 PMC3184548

[B77] Jia R Li C McCoy JP Deng CX Zheng ZM SRp20 is a proto-oncogene critical for cell proliferation and tumor induction and maintenance Int J Biol Sci 2010 6 806 826 21179588 10.7150/ijbs.6.806 PMC3005347

[B78] He X Arslan AD Pool MD Ho TT Darcy KM Coon JS Beck WT Knockdown of splicing factor SRp20 causes apoptosis in ovarian cancer cells and its expression is associated with malignancy of epithelial ovarian cancer Oncogene 2011 30 356 365 10.1038/onc.2010.426 20856201 PMC3010329

